# Incorporating ultrafiltration into Protein A membrane chromatography as a strategy to reduce elution volume and buffer consumption

**DOI:** 10.14440/jbm.2025.0109

**Published:** 2025-04-10

**Authors:** Gaoya Yuan, Meng Qu, Xudong Zhang, Yifeng Li

**Affiliations:** Downstream Process Development (DSPD), WuXi Biologics, Waigaoqiao Free Trade Zone, Shanghai 200131, China

**Keywords:** Elution volume, Buffer consumption, Dead volume, Protein A membrane, Ultrafiltration

## Abstract

**Background:**

Protein A chromatography is widely used for antibody purification. With conventional packed-bed columns, mass transfer within resin beads is diffusion-limited, entailing long residence time to achieve high binding capacities. Recently, several vendors have introduced Protein A membranes as alternatives to traditional Protein A resins/columns. These membranes feature open pore structures that facilitate the convective transport of protein molecules, enabling high binding capacities within significantly shorter residence time. The use of Protein A membranes can improve throughput, eliminate the need for column packing, and reduce cost. These advantages notwithstanding, Protein A membranes present certain drawbacks. A major limitation is their high dead volume-to-stationary phase ratio, which leads to larger elution volumes compared to their conventional counterparts. This results in significant eluate dilution and increased buffer consumption.

**Objective:**

In the current study, we aimed to demonstrate that ultrafiltration (UF), when used in combination with Protein A membrane chromatography, can address these limitations by allowing eluate concentration and buffer reuse.

**Methods:**

A laboratory model of UF integrated Protein A membrane was set up to test the feasibility and effectiveness of the proposed strategy.

**Results:**

Integrated UF effectively concentrated Protein A membrane eluate to a concentration comparable to that of Protein A column eluate. In addition, reuse of pH-adjusted UF filtrate as elution buffer reduces buffer consumption by 50%.

**Conclusion:**

UF integration is an effective solution for addressing the problem of increased elution volume and buffer consumption associated with Protein A membrane.

## 1. Introduction

Protein A chromatography is extensively used for product capture in the downstream processing of monoclonal antibodies, bispecific antibodies, and Fc-fusion proteins. When performed using packed-bed columns, mass transfer is limited by slow intraparticle diffusion, necessitating long residence time (typically 5 – 6 min) to attain high binding capacities. This need for extended residence time results in prolonged processing times and reduced productivity.^1^ The diffusional and flow limitations associated with resin beads can be overcome using membrane-based media.^2^,^3^ Unlike resin beads, membranes possess open pore structures that allow convective flow to rapidly deliver solute molecules to the ligand. Recently, several manufacturers – including Sartorius, Cytiva, and Gore – have launched Protein A membrane products (Sartobind Protein A/Rapid A, HiTrap Fibro PrismA, and Protein Capture Device, respectively).^4^-^8^

In these membranes, convective transport significantly reduces mass transfer resistance, enabling binding capacities comparable to those of conventional resins in much shorter residence time (measured in seconds).^4^ For example, the HiTrap Fibro PrismA achieves a binding capacity of 70 mg/mL in a residence time of just 5 s.^7^ As a result, the cycle time can be reduced from several hours to <10 min when Protein A resins are replaced with Protein A membranes. These membranes can typically be reused for up to 200 cycles, and the short cycle time allows for full utilization of their operational lifespan within single batch purification. Therefore, the amount of chromatography material needed can be 10 times less than that of resin-based systems, significantly lowering production costs.^9^

Importantly, studies did not show significant differences in product quality or yield between samples processed using Protein A resins and those processed with Protein A membranes.^5^,^6^,^9^,^10^ In addition, Protein A membranes demonstrated good scalability.^6^,^9^

Despite these advantages, Protein A membranes also have certain drawbacks. Several studies reported that a notable issue is a significantly larger elution volume in comparison to that of equivalently sized Protein A columns.^4^,^5^,^9^ We observed the same phenomenon during our use of Protein A membranes for product capture. This issue arises from the large dead volume-to-stationary phase ratio inherent to membrane design, which causes poor flow distribution, resulting in early product breakthrough and peak broadening during elution.^4^,^5^,^9^,^11^

Large elution volumes are particularly undesirable in large-scale manufacturing, as they can pose facility fit challenges – especially considering that one of the primary goals of the Protein A capture step is to reduce the handling volume. In addition, larger elution volumes increase buffer consumption, thereby raising production costs. Addressing this issue is therefore critical for enabling more widespread application of Protein A membranes in bioprocessing.

In the current work, we presented a practical solution to this problem by integrating Protein A membrane chromatography with ultrafiltration (UF). While UF enables the concentration of the Protein A membrane eluate, the resulting filtrate (upon pH adjustment) can be reused as an elution buffer. This combined strategy simultaneously addresses the issues of excessive elution volume and high buffer consumption associated with Protein A membrane use.

## 2. Materials and methods

### 2.1. Materials

Sodium acetate trihydrate, sodium chloride, sodium hydroxide, and tris(hydroxymethyl)aminomethane (Tris) were purchased from Merck (Germany). Acetic acid was procured from J.T. Baker (United States America [USA]). The Sartobind Rapid A Protein A membrane was obtained from Sartorius (Germany). The UF membrane, Pellicon 3 Cassette with Biomax 30 kDa Membrane (A screen, 88 cm^2^), was from Millipore (USA). MabSelect PrismA was bought from Cytiva (Sweden). The Vantage L Laboratory Column VL (11 × 250 mm) came from Millipore (USA), and the Protein BEH SEC Column (4.6 × 150 mm) from Waters (USA). The WXB CHO-K1 HCP ELISA Kit was sourced from WuXi Biologics (China).

The mAb used in this study was expressed in stably-transfected CHO-K1 cells cultured in HyClone ActiPro culture medium supplemented with Cell Boost 7a and 7b (both from Cytiva, Sweden). The cell culture was maintained for 14 days before harvest. Additional information regarding the mAb and the corresponding culture harvest used as the Protein A column/membrane feed are provided in Table 1.

**Table 1:** The monoclonal antibody (mAb) and the corresponding culture harvest

mAb type	Isoelectric point	Viable cell density (10^6^/mL)	Viability (%)	Titer (g/L)
IgG1	9.1	9.6	82	4.52

### 2.2. Equipment

An AKTA Pure 150 system installed with Unicorn software version 7.8 (Cytiva, Sweden) was employed for chromatography. pH and conductivity were measured using a SevenExcellence S470 pH/Conductivity meter (Mettler-Toledo, USA). Protein concentration was determined on a NanoDrop 2000 spectrophotometer (Thermo Fisher Scientific, USA). Magnetic stirrers, including the PC-410D from Corning (USA) and the MIDI MR1 digital from IKA (Germany), were utilized for sample and buffer mixing. The BT100-2J peristaltic pump from LongerPump (China) was used for UF. An ACQUITY UPLC H-Class PLUS Bio System (Waters, USA) was used for size-exclusion chromatography-ultra-high performance liquid chromatography (SEC-UPLC). Host cell protein (HCP) quantitation results were read by an Infinite 200 PRO plate reader from Tecan (Switzerland).

### 2.3. Protein A column chromatography

MabSelect PrismA resin was used for Protein A column chromatography. The protocol is summarized in Table 2. A column (1.1 cm diameter) was packed with MabSelect PrismA to a bed height of 17.7 cm, resulting in a column volume (CV) of approximately 16.8 mL. The column was loaded at a protein density of 45 mg/mL of resin. After loading, the column was washed sequentially with (i) 50 mM Tris, acetic acid (HAc), 150 mM sodium chloride (NaCl), pH 7.4; (ii) 50 mM NaAc-HAc, 1 M NaCl, pH 5.5, and (iii) 30 mM NaAc-HAc, pH 5.5, each for 3 CV. Elution was carried out using 30 mM NaAc-HAc, pH 3.6. The system was run at a flow rate of 212 cm/h, corresponding to a 5-min residence time).

**Table 2:** Protocol for Protein A column chromatography

Step	Buffer/solution	CV	Flow rate (CV/min)	Time^a^ (min)
Rinse	50 mM Tris, HAc, 150 mM NaCl, pH 7.4	2	0.2	10.0
Sanitization	0.5 M NaOH	3	0.2	15.0
Equilibration	50 mM Tris, HAc, 150 mM NaCl, pH 7.4	3	0.2	15.0
Load	Clarified culture harvest	13	0.2	64.3
Wash 1	50 mM Tris, HAc, 150 mM NaCl, pH 7.4	3	0.2	15.0
Wash 2	50 mM NaAc-HAc, 1 M NaCl, pH 5.5	3	0.2	15.0
Wash 3	30 mM NaAc-HAc, pH 5.5	3	0.2	15.0
Elution	30 mM NaAc-HAc, pH 3.6	3	0.2	15.0
Strip	0.12 M HAc	3	0.2	15.0
Rinse	50 mM Tris, HAc, 150 mM NaCl, pH 7.4	2	0.2	10.0
Sanitization	0.5 M NaOH	3	0.2	15.0
Rinse	50 mM Tris, HAc, 150 mM NaCl, pH 7.4	2	0.2	10.0
Storage	20% EtOH	3	0.2	15.0

Note:

a Total cycle time was 229.3 min.

Abbreviations: CV: Column volume; HAc: Acetic acid; NaCl Sodium chloride; Tris: Tris (hydroxymethyl) aminomethane.

### 2.4. Protein A membrane chromatography

Sartobind Rapid A was used for Protein A membrane chromatography by following the protocol listed in Table 3. The membrane volume (MV) was 10 mL, and loading was performed at a density of 30 mg of protein per mL of membrane. After loading, the membrane was washed sequentially with 50 mM Tris, HAc, 0.5 M NaCl, pH 7.4, and 30 mM NaAc-HAc, pH 5.5, using 10 MV of each buffer. Elution was conducted with 30 mM NaAc-HAc, pH 3.6, over 11.4 MV. The system was operated at a flow rate of 5 MV/min (residence time: 12 s) for the loading and elution steps and 10 MV/min for all the other steps.

**Table 3:** Protocol for Protein A membrane chromatography

Step	Buffer/solution	MV	Flow rate (MV/min)	Time^a^ (min)
Rinse	50 mM Tris, HAc, 150 mM NaCl, pH 7.4	10	10	1.0
Sanitization	0.2 M NaOH	10	10	1.0
Equilibration	50 mM Tris, HAc, 150 mM NaCl, pH 7.4	10	10	1.0
Load	Clarified culture harvest	8.6	5	1.3
Wash 1	50 mM Tris, HAc, 0.5 M NaCl, pH 7.4	10	10	1.0
Wash 2	30 mM NaAc-HAc, pH 5.5	10	10	1.0
Elution	30 mM NaAc-HAc, pH 3.6	11.4	5	2.4
Strip	0.12 M HAc	10	10	1.0

Note:

a Total cycle time lasted 9.7 min.

Abbreviations: HAc: Acetic acid; NaCl: Sodium chloride; Tris: Tris (hydroxymethyl) aminomethane.

### 2.5. UF in combination with Protein A membrane chromatography

A UF system was incorporated into the Protein A membrane chromatographic process using two 88 cm^2^ UF membranes. Before use, the UF membranes were flushed with purified water, sanitized with 1 M NaOH, rinsed again with purified water, and equilibrated with 30 mM NaAc-HAc buffer at pH 4.1. The eluate from each Protein A chromatographic cycle was collected into Tank A, which served as both the feed and the retentate collection tank for the UF system. The UF process was operated at a feed flux of 60 – 75 L/m^2^/h, selected to align with Protein A membrane elution flow rate (approximately 7 mL/min), and a transmembrane pressure of 1.6 – 2.9 psi. The UF permeate was collected into Tank B, whose pH was adjusted to 3.6 using 1 M HAc through in-line conditioning with an AKTA Pure 150M system. The pH-adjusted UF filtrate was subsequently transferred to the Protein A elution buffer tank (Tank C), from which it was reused for membrane elution.

### 2.6. Size-exclusion chromatography-ultra-high performance liquid chromatography

Size-exclusion chromatography-ultra-high performance liquid chromatography was performed on an ACQUITY UPLC H-Class PLUS Bio System equipped with an ACQUITY UPLC Protein BEH SEC Column (4.6 × 150 mm). A 10 μg sample was injected per run. The mobile phase consisted of 50 mM sodium phosphate and 300 mM sodium chloride at pH 6.8. Isocratic elution was carried out over a period of 8 min at a flow rate of 0.4 mL/min. Protein elution was monitored using ultraviolet absorbance at 280 nm.

### 2.7. Host cell protein quantification

Host cell protein levels were quantified using the WXB CHO-K1 HCP ELISA Kit and following the manufacturer’s instructions. The detection range was 3 – 100 ng/mL. Serial dilutions of the samples were prepared to ensure measurements fell within the calibration range. Absorbance was measured at the wavelength of 450 nm, with 650 nm used as the reference, by utilizing an Infinite 200 PRO plate reader.

## 3. Results and discussion

### 3.1. Replacing the Protein A column with a Protein A membrane increases elution volume and buffer consumption

Protein A membranes, as alternatives to conventional Protein A resins/columns, enable high binding capacities to be achieved at significantly higher flow rates, as membrane-based binding is less dependent on residence time. As shown in Tables 2 and 3, the total cycle time for Protein A column and Protein A membrane chromatography is 229.3 and 9.7 min, respectively, regardless of whether CV or MV is used. Consequently, a given volume of culture harvest can be processed in approximately the same amount of time using a membrane with a much smaller volume than the column, thereby reducing resin-related costs.

**Figure 1:**
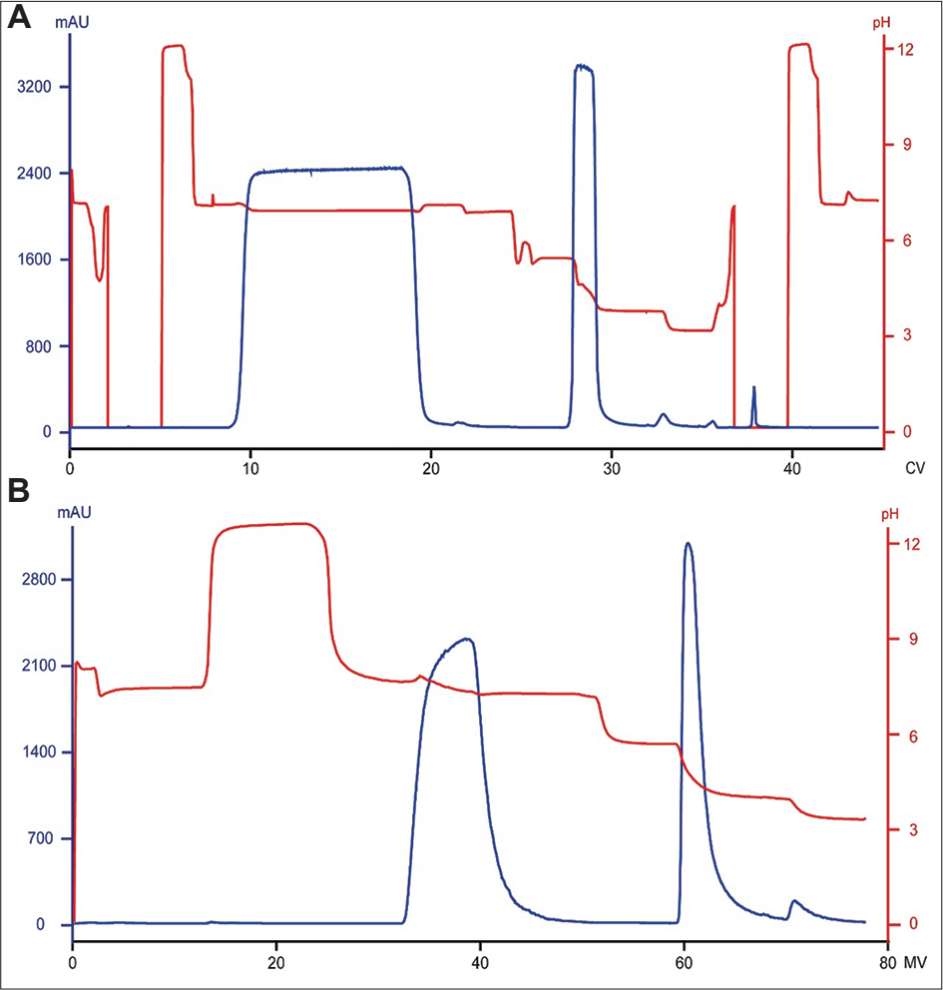
Chromatograms of runs conducted with (A) Protein A column and (B) Protein A membrane, both using stepwise pH gradient elution Abbreviations: CV: Column volume; MV: Membrane volume.

When Protein A column chromatography is conducted in a 5-min residence time, the elution volume typically ranges from 1.5 to 2.2 CV (with 3 CV of elution buffer applied), depending on the elution pH and collection criteria. For example, in the run corresponding to Figure 1A, an elution volume of 2.1 CV was observed at pH 3.6 with a collection range of 50 – 50 mAU/mm. In contrast, at the manufacturer-recommended flow rates for Protein A membrane chromatography (i.e., 5 MV/min for loading and elution; 10 MV/min for all other steps), elution volumes range from 6 to 7 MV at identical elution pH and under the same collection conditions (Figure 1B). For a culture harvest with a titer of 4.52 mg/mL, the resulting eluate concentrations from the Protein A column and Protein A membrane were 20 – 26 mg/mL and 3.7 – 3.9 mg/mL, respectively. This finding indicates that the elution volume from the Protein A membrane is approximately 5 – 6-fold greater than that of the column. Such an increase necessitates a larger collection tank, which poses challenges for large-scale manufacturing. Additionally, the increased elution volume directly correlates with higher buffer consumption. In stepwise pH gradient elution, the elution buffer volumes required were 3 CV for the Protein A column and 11.4 MV for the membrane.

A previous study demonstrated that the elution volume of the Sartobind Protein A membrane was affected by the elution flow rate, with the volume roughly doubling as the flow rate increased from 0.5 to 5 MV/min (corresponding to a decrease in residence time from 120 to 12 s).^4^ In the current work, we also evaluated the impact of elution flow rate on elution volume. In addition to the vendor-recommended flow rate of 5 MV/min (12-s residence time), we tested three additional flow rates: 2, 1, and 0.5 MV/min (corresponding to residence time of 30, 60, and 120 s, respectively). Our results showed that elution volume varied only modestly with flow rate. Specifically, at flow rates of 5, 2, 1, and 0.5 MV/min, the corresponding elution volumes were 6.7, 8.6, 7.3, and 7.2 MV, respectively.

Interestingly, the maximum elution volume (i.e., 8.6 MV) was observed at 2 MV/min. As lower flow rates help mitigate poor flow distribution, marginally reduced elution volumes were observed when residence time was increased (i.e., 7.3 and 7.2 MV). At the higher flow rate (i.e., 5 MV/min), flow distribution was further compromised, which would typically increase the elution volume. However, under these conditions, elution was incomplete, resulting in a lower elution volume (i.e., 6.7 MV), but at the cost of product yield.

It is also worth noting that the membranes used in the current and previous studies were different: Sartobind Rapid A was employed in the present work and Sartobind Protein A in the previous studies. Although both are manufactured by Sartorius, they differ significantly in performance and intended use. Sartobind Rapid A offers a binding capacity >45 mg/mL and is suitable for laboratory, pilot-scale, and good manufacturing practice (GMP) production. In contrast, Sartobind Protein A has a binding capacity below 8 mg/mL and is designed for laboratory-scale applications only. These differences likely account for the discrepancies observed between the two studies.

### 3.2. UF overcomes the drawbacks associated with Protein A membranes

**Figure 2:**
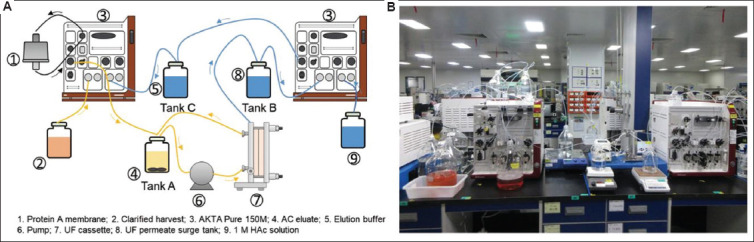
(A) Schematic representation and (B) corresponding laboratory-scale setup for Protein A membrane chromatography combined with UF. In this design, the Protein A membrane eluate is simultaneously and continuously concentrated through UF. The flow pathway is indicated, and key equipment is labeled. Upon pH adjustment, the UF permeate is reused as an elution buffer. Collection tanks for the membrane eluate (Tank A), UF permeate (Tank B), and elution buffer (Tank C) also serve as surge tanks to maintain synchronization during minor fluctuations. Abbreviations: AC: Affinity chromatography; HAc: Acetic acid; UF: Ultrafiltration.

Increased elution volume poses challenges for large-scale manufacturing and this limitation has to be addressed for broader adoption of Protein A membrane chromatography. UF, a widely used technique for protein concentration, was therefore integrated into the Protein A membrane chromatography to allow for simultaneous concentration during elution. Furthermore, since the UF permeate is essentially an elution buffer with a slightly increased pH, we hypothesized that, by means of appropriate pH adjustment, it could be reused as an elution buffer. This approach not only addresses the issue of high elution volume but also significantly reduces elution buffer consumption. A schematic representation and the corresponding laboratory-scale setup of the integrated Protein A membrane chromatography–UF system are shown in Figure 2A and B, respectively.

**Figure 3:**
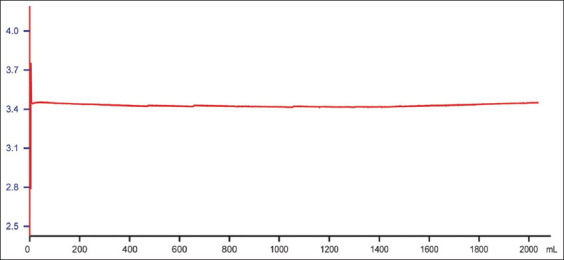
pH curve of the adjusted UF filtrate. The relatively stable pH of the Protein A membrane eluate allows for accurate adjustment of UF filtrate to the desired value (3.6) by adding a consistent volume of acid. Abbreviation: UF: Ultrafiltration.

**Figure 4:**
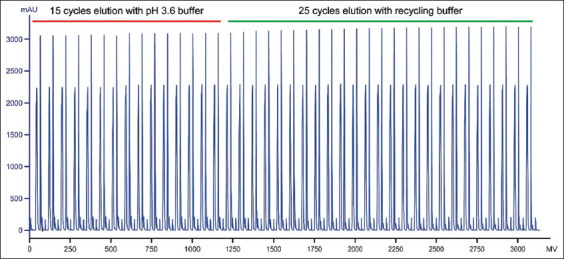
Ultraviolet chromatograms of 40 consecutive Protein A membrane chromatography cycles with integrated ultrafiltration. For the last 25 cycles, a portion of the elution buffer in each cycle was derived from pH-adjusted UF filtrate. Abbreviation: UF: Ultrafiltration.

UF-mediated protein concentration is relatively straightforward. To reuse the UF filtrate as an elution buffer, its pH must be precisely adjusted to the targeted value. We observed that the eluate pH remained relatively stable, enabling consistent pH control of the UF filtrate by adding a fixed amount of acid per unit volume. The pH adjustment curve is shown in Figure 3, indicating that the adjusted filtrate pH remained tightly centered around the target value of 3.6. According to the protocol shown in Table 3, using a 10 mL Protein A membrane, the total volume of elution buffer required for 40 cycles is approximately 4.6 L (10 mL × 11.4 × 40 = 4,560 mL). Using the laboratory setup shown in Figure 2B, 40 consecutive cycles were conducted (Figure 4 for chromatograms). The integrated UF system allowed eluate concentration to reach 25 mg/mL, comparable to that of Protein A column eluate. Although higher concentrations could be achieved, they were deemed unnecessary at this stage.

Importantly, reusing the pH-adjusted UF filtrate as elution buffer reduced the total elution buffer requirement to approximately 2.5 L—a virtually 50% reduction in comparison to the standard process without buffer recycling. As shown in Figure 4, the UV chromatograms of all 40 cycles were highly consistent, suggesting stable system performance and confirming the effectiveness of the pH-adjusted UF filtrate as an elution buffer. Furthermore, key quality attributes – such as SEC-UPLC purity and HCP levels – were comparable between runs conducted with and without UF (Table 4), supporting the feasibility of using this strategy for both elution volume control and buffer conservation.

**Table 4:** Quality data for Protein A membrane eluates obtained with and without ultrafiltration

Sample description	SEC-UPLC (%)	HCP (ng/mg)
Protein A membrane eluate (without UF)^a^	100	606
Protein A membrane eluate (with UF)^b^	99.9	918^c^

Notes:

a Sample from a single cycle.

b Sample from a pooled eluate of 40 cycles.

c HCP concentration in the UF filtrate was approximately 50 ng/mL, suggesting the limited passage of HCPs through the membrane and minimal recycling back into the system.

Abbreviations: HCP: Host cell protein; SEC-UPLC: Size-exclusion chromatography-ultra-high performance liquid chromatography.

Even if HCPs in the Protein A membrane eluate could pass through the membrane and re-enter the system, this supposedly would not affect final HCP levels, as no new HCPs were introduced. Consistently, the HCP level in the UF-integrated process (918 ng/mg) was only modestly higher than that in the non-UF process (606 ng/mg). This slight difference may reflect small variations in buffer composition, as the UF-integrated process reuses pH-adjusted UF filtrate as an elution buffer.

To assess the potential for scale-up, we made a theoretical comparison of Protein A column chromatography and Protein A membrane chromatography (with and without UF), assuming the need to process 2,000 L of culture harvest (titer: ~4.52 mg/mL). The column process uses 67 L of resin (column dimension: 60 × 23.7 cm), and the membrane process uses 1.6 L of membrane material (two 0.8 L membranes in parallel). In both cases, processing is completed within 2 days. The loading densities were 45 mg/mL for the column and 30 mg/mL for the membrane. The number of cycles required were 3 for the column and 189 for the membrane.

According to the protocols shown in Tables 2 and 3, the total elution buffer required was 603 L for the column (67 L × 3 CV × 3 cycles = 603 L) and 3,447 L for the membrane without UF (1.6 L × 11.4 MV × 189 cycles). According to our laboratory-scale results, integrating UF and reusing pH-adjusted filtrate reduced buffer consumption by approximately 50%, while still achieving eluate concentrations and volumes comparable to those from column chromatography.

## 4. Conclusion

Protein A membranes represent a promising alternative to traditional resin-based Protein A columns. Replacing Protein A columns with Protein A membranes substantially improve throughput and cost efficiency. However, one major drawback of Protein A membranes is the significantly increased elution volume and buffer consumption, which can present operational challenges and incur added costs in large-scale manufacturing. In the current work, we demonstrated that integrating UF with Protein A membrane chromatography offers a practical solution to this problem. In addition to concentrating the eluate, the UF filtrate – after appropriate pH adjustment – can be reused as an elution buffer. This design effectively reduces both the elution volume and buffer consumption.

As demonstrated with a laboratory-scale model, the integrated UF system accomplished eluate volumes and concentrations similar to those from column chromatography, while reducing elution buffer usage by approximately 50% relative to processes without UF filtrate reuse. We also evaluated the use of single-pass tangential flow filtration (SPTFF) with a Pall membrane (186 cm^2^ × 6, configured in 3 stages-in-series) for the same purpose. While SPTFF achieved a concentration factor of 5, its feed flux (~290 mL/min) did not match the relatively low flow rate of Protein A membrane elution (7 mL/min), rendering it unsuitable for the current setup. Nevertheless, SPTFF may be well-suited for larger Protein A membranes used in pilot- and GMP-scale manufacturing.

In conclusion, the strategy presented in the current work provides a feasible solution to the challenges of increased elution volume and buffer consumption associated with Protein A membrane chromatography. We believe this approach can help facilitate the broader application of Protein A membranes by mitigating their current drawbacks and better leveraging their inherent advantages.

## Data Availability

The data and supporting information are available within the article.
